# Physical constraints during Snowball Earth drive the evolution of multicellularity

**DOI:** 10.1098/rspb.2023.2767

**Published:** 2024-06-26

**Authors:** William W. Crockett, Jack O. Shaw, Carl Simpson, Christopher P. Kempes

**Affiliations:** ^1^ Department of Biology, Massachusetts Institute of Technology, Cambridge, MA 02139, USA; ^2^ Santa Fe Institute, Santa Fe, NM 87501, USA; ^3^ Department of Geological Sciences and University of Colorado Museum of Natural History, University of Colorado, Boulder, CO 80309, USA

**Keywords:** multicellularity, Neoproterozoic, major evolutionary transitions, biophysical constraints, metabolic theory of ecology, physical modelling

## Abstract

Molecular and fossil evidence suggests that complex eukaryotic multicellularity evolved during the late Neoproterozoic era, coincident with Snowball Earth glaciations, where ice sheets covered most of the globe. During this period, environmental conditions—such as seawater temperature and the availability of photosynthetically active light in the oceans–likely changed dramatically. Such changes would have had significant effects on both resource availability and optimal phenotypes. Here, we construct and apply mechanistic models to explore (i) how environmental changes during Snowball Earth and biophysical constraints generated selective pressures, and (ii) how these pressures may have had differential effects on organisms with different forms of biological organization. By testing a series of alternative—and commonly debated—hypotheses, we demonstrate how multicellularity was likely acquired differently in eukaryotes and prokaryotes owing to selective differences on their size due to the biophysical and metabolic regimes they inhabit: decreasing temperatures and resource availability instigated by the onset of glaciations generated selective pressures towards smaller sizes in organisms in the diffusive regime and towards larger sizes in motile heterotrophs. These results suggest that changing environmental conditions during Snowball Earth glaciations gave multicellular eukaryotes an evolutionary advantage, paving the way for the complex multicellular lineages that followed.

## 1. Introduction

A fundamental focus of biology is understanding the vast range of body sizes and the associated diversity in the number of levels of hierarchical organization [1,2]. Each new level of organization is typically associated with a major event in evolutionary history that changed the state of the evolutionary game. By adding a new hierarchical level to the organization of organisms, these major transitions in individuality added new niches to the ecosystem (e.g. trophic) and introduced new phenotypes. Such transitions include the origin of cells, eukaryotes, multicellularity, and colonial and social organisms. The insight that these transitions share evolutionary processes involved in the emergence of a new level of organization has proven to be a powerful research programme (see [1,3–5] for comprehensive reviews of the topic).

However, it is challenging to understand certain transitions, such as multicellularity, because of the large number of independent origins, the fact that eukaryotes and prokaryotes both evolve multicellular forms, and the lack of substantial fossil and molecular evidence [6,7]. The evolution of multicellularity stands as one of the most pivotal milestones in the history of life on Earth as it revolutionized biological organization and paved the way for the diversity of macro-scale organisms we observe today. Its emergence allowed specialized cells to cooperate, leading to the development of complex tissues, organs, and organ systems. This enhanced complexity further facilitated the evolution of complex organisms with more sophisticated behaviours enabling adaptation to a wide range of environments and the exploitation of new ecological niches and new biological scales. Multicellularity laid the foundation for the diverse and interconnected web of life that shapes our planet’s ecosystems today.

Fossil and molecular evidence indicates that complex multicellularity originated and proliferated during the Neoproterozoic era (1000–541 Ma) [8,9]. Previous work commonly proposed that this evolution was connected to an increase in oxygen levels that removed a physical constraint on size. However, recent work suggests that sponges, a likely morphology for the last common metazoan ancestor, can survive oxygen levels as low as those present during the Neoproterozoic era [10], suggesting that low oxygen levels may not have been a physical constraint preventing the emergence of multicellular eukaryotes. Furthermore, other work suggests that the evolution of more complex eukaryotes, including multicellular organisms, could have led to ocean oxygenation [11] (as opposed to the other way around), and we know that multicellular eukaryotes can cope with low oxygen given that it is likely that the sea floor was anoxic when the first undisputed metazoan fossils appeared in deep water [12–14]. If the appearance of multicellularity was not caused by changing oxygen levels, an alternative mechanism for why multicellular eukaryotes emerged during this period is needed.

Extreme glaciations during the Cryogenian period (approx. 720−635 Ma), a phenomenon commonly referred to as Snowball Earth, led to a radical transformation of the Earth’s climate and oceans [15]. Across two major glaciations, lasting almost 50 Myr, glaciers appear to have reached the Equator, although there is still debate over the extent of coverage [16,17]. The global glaciations resulted in the widespread freezing of the planet’s surface, severely restricting the availability of light and nutrients to depths below. Prior to Snowball Earth, simulations suggest the ocean was relatively warm, with surface water temperatures reaching 30°C at the Equator [18]. However, depending on the severity of glaciations, temperatures likely dropped to between −4 and 4°C [17,19].

Given that such extreme conditions persisted for tens of millions of years, it is important to understand how these conditions affected the evolutionary trajectories of existing organisms, which requires a greater understanding of the broader environmental and ecological changes that occurred. Molecular clock estimates show significant bottlenecks in populations of autotrophs during this period [20], but others report the maintenance of an active, biologically mediated, nitrogen cycle [21]. Whether organisms were confined to small environmental niches on top of the ice or there were areas of open ocean under ‘slush-ball’ conditions, both autotrophs and heterotrophs would have had to contend with lower temperature, light availability and nutrient concentrations [17]. The decrease in temperature would have slowed down metabolism and diffusion rates, further decreasing primary productivity.

Despite evidence of profound environmental and ecological change, fossil evidence does not indicate any significant extinctions [22,23]. One potential means of success in these conditions may have been found in the formation of cooperative groups of cells in some lineages, which then could have led to the emergence of multicellular life. Interestingly, an increased abundance of eukaryotes and an increase in mean organism size have been proposed as a mechanism for the end of the Neoproterozoic glacial events, owing to an increase in organic matter sinking rates [24].

Recent work [25] suggests that the long-term loss of low-viscosity environments, instigated by decreasing ocean temperatures during the Cryogenian, generated selective pressures towards multicellularity in eukaryotes. This work suggests that adaptation to environmental conditions led to larger sizes and speeds only accessible through multicellularity to exploit limited resources and satisfy metabolic needs during Snowball Earth’s high-viscosity regimes. Following the cessation of glaciation and the return of low-viscosity environments, these newly evolved multicellular taxa remained and proliferated.

Beyond the viscosity shifts associated with the much lower temperatures of Snowball Earth there are many other physical, physiological and ecological changes expected during this interval (see e.g. [17,21,26,27]). For example, the accumulation of significant sea ice likely decreased light flux to the ocean and decreased the terrestrial nutrient run-off [16,17]. Ecological and biogeochemical features associated with sinking, remineralization, predation and the size distribution of organisms are all also expected to shift in this new environment.

For an organism to survive it must be able to access enough nutrients to satisfy metabolic demands. Several factors can be altered and integrated to allow an organism to increase nutrient capture, including metabolic rate, motility and size. Given the existence of numerous optima, the specific combination of changes to metabolic rate, motility and size is less important than the first-order need to acquire nutrients.

Because of the multiple contemporaneous origins of eukaryotic multicellularity an environmental driver is likely. However, an environmental driver cannot be universal because only a few of the many co-occurring eukaryotic lineages evolved multicellularity, such that the driver must also sort between adaptive strategies. An answer may be found if there are competing biophysical aspects that share a common cause. Cold conditions during Snowball Earth may provide such a cause, with effects on viscosity, diffusivity and metabolic rates that led to complex trade-offs.

**Figure 1.**
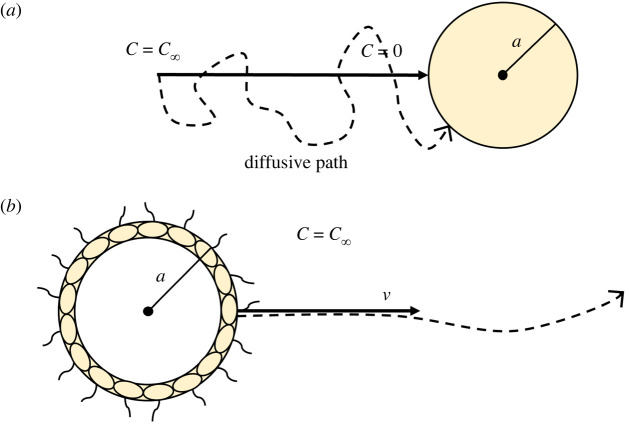
(*a*) Non-motile diffusive cell. The spherical cell takes in all nutrients at the cell’s surface (*C* = 0), causing chemical resources (e.g. glucose) to diffuse towards the cell from far away (*C* = *C*_∞_). (*b*) Motile choanoblastula. The organism is hollow with an outer radius *a*, and swims at a velocity *v*. The organism’s motility means it travels ballistically relative to its prey. Resource concentration is assumed to be constant (*C* = *C*_∞_).

This paper presents analyses of mechanistic models for exploring interactions between the environmental changes associated with Snowball Earth, physical constraints on biological processes, and differential selective pressures between single-celled and simple multicellular organisms. First, we describe a global productivity model that suggests Snowball Earth’s changes in temperature and light availability generated a significant decrease in primary production. Second, based on this insight, we compare two models that describe how organisms with different biological organizations—a non-motile unicellular organism relying on diffusion (figure 1*a*) and a simple motile multicellular organism—are affected by the environmental changes predicted during Snowball Earth.

For our multicellular organism, we model a hypothetical and idealized ‘choanoblastula’ (figure 1*b*). The choanoblastula is heterotrophic, motile and composed of a hollow sphere of cells, such that it has similar morphology and physiology to the green algae genus *Volvox*, except that it does not photosynthesize. Something akin to this model organism may have existed during the Cryogenian, but would have been displaced by descendant lineages (e.g. Metazoa).

Our results suggest differential responses to selective pressures: (i) for organisms operating in the diffusive regime, decreasing temperature and resource availability leads to a decrease in organismal size; and (ii) for motile heterotrophs with a simple multicellular morphology, environmental changes accompanying Snowball Earth selected for larger organisms.

## 2. Methods

### (a) Global productivity model

To understand the impacts that Snowball Earth had on eukaryotes and early Metazoa, it is crucial to understand how the environmental changes impacted the broader ecosystem. A simple method to estimate the magnitude of these changes is to calculate the net primary productivity (NPP) as a function of temperature and intensity of photosynthetically active radiation (PAR) [28]:

2.1 $$\mathrm{NPP}=\frac{1}{V}\sum_{i=1}^{n_{a}}\epsilon P_{i},$$

where *V* is the volume of water, *n*_a_ is the number of autotrophic cells, ϵ is the efficiency of production of organic matter and *P*_*i*_ is the productivity of each autotrophic cell. The productivity of each autotroph can be modelled as a function of it is metabolic rate and PAR. The metabolic rate is modelled using the metabolic theory of ecology (MTE) [29,58], which relates metabolism (*B*) to temperature (*T*) and organism mass (*M*_*i*_):

2.2 $$B=b_{0}\;e^{-E_{a}/kT}M_{i}^{\alpha },$$

where *E*_a_ is the average activation energy of metabolic reactions, *b*_0_ is a constant, *k* is Boltzmann’s constant and *α* is a power-law scaling term. The scaling term *α* is normally assigned a value of 3/4 for multicellular organisms, and 1 for single-celled eukaryotes [30,31].

Productivity’s dependence on light intensity (*I*) is given by a Monod equation [32], where *K_I_* is the half-saturating term. Combining the dependence of productivity on metabolic rate and light intensity results in the following expression [28]:

2.3 $$P_{i}=p_{0}\;e^{-E_{a}/kT}\frac{I}{I+K_{I}}M_{i}^{\alpha },$$

where *p*_0_ is a constant.

To model *n*_a_, the steady-state biomass model in [33] is employed. Assuming constant cell size, this model calculates the supported biomass under given nutrient flux conditions, allowing us to solve for the population carrying capacity for a given set of environmental conditions.

### (b) Uptake–metabolism energy balance

An energy balance was used to model the impact of changing temperature and resource concentration on organisms, where the rate of energetic resource uptake (*U*) must be greater than or equal to the rate of energy use in the organism’s metabolism (*B*):

2.4 U≥B.

To understand how environmental changes altered optimal phenotypes, resource uptake and metabolism can be modelled as functions of temperature, resource concentration and organismal traits (which are assumed to be generated from body size). Both rates depend on specific resource acquisition strategies and organism morphologies, two of which we explore here.

#### (i) The non-motile diffusive cell

The modelled organism was inspired by smaller prokaryotes, with the following traits: single-celled, non-motile and reliant upon diffusion for uptake (figure 1*a*). Assuming that the cell takes up all resources at its surface, and that resource concentration approaches a constant (*C*_∞_) far away from the cell, we can solve the diffusion equation to obtain an equation for resource concentration

2.5 $$C=C_{\infty }\left(1-\frac{a}{r}\right),$$

where *a* is the radius of the cell and *C* is the nutrient concentration at some distance *r* from the cell’s centre (see electronic supplementary material for detailed derivation). The cell’s total resource influx can be determined by applying Fick’s Law of Diffusion [34] to calculate flux density and integrating it across the cell’s surface [35]:

2.6 $$U=4\pi DaC_{\infty }.$$

Here, *D* is the diffusivity of the resource, which can be defined by the Stokes–Einstein equation [36]. Viscosity (*η*), can be modelled as a function of temperature using the Vogel–Fulcher–Tammann (VFT) equation [37]. Diffusivity is inversely proportional to this viscosity. By incorporating these physical models into the uptake model (equation (2.6)) resource uptake for the diffusive cell is modelled as a function of temperature, resource concentration and cell size:

2.7 $$U(T,C_{\infty },a)=\frac{2}{3}\frac{kT}{\eta_{0}R}\;e^{-A/\left(T-C\right)}aC_{\infty }$$

Equation (2.2) is used to model the metabolic rate of the diffusive cell [29]. Also, the conversion between volume and mass is approximated using a constant cell density. Using these definitions for resource uptake and metabolic rate in equation (2.4) and solving the inequality for organism radius (*a*) results in the model for the maximum diffusive cell size as a function of temperature and resource concentration [58]:

2.8 $$a\leq {\left(\frac{2}{3}\frac{kT}{\eta_{0}r}e^{-A/(T-C)}\frac{C_{\infty }}{B_{0}}e^{-E_{a}(T-T_{0})/kTT_{0}}\right)}^{1/(3\alpha -1)}.$$

#### (ii) The motile choanoblastula

The choanoblastula employs a different uptake strategy, and its morphology leads to a different mass–metabolism scaling relation. The resource uptake rate is based on ballistic velocity of the organism, and its metabolism is based on the MTE and an additional motility cost.

Owing to the relative difference in velocity that arises from the choanoblastula’s motility, its uptake is ballistic rather than diffusive (figure 1) [38,39]. In this case, the choanoblastula is colliding with its resource, causing resource uptake to scale with its cross-sectional area [40]:

2.9 $$U=\pi a^{2}vC_{\infty },$$

where *v* is the velocity of the choanoblastula relative to the resource. The velocity scales with organism radius and the viscosity of the surrounding fluid [41]. This is summarized in the generalized model [25]

2.10 $$v=\beta a^{b}\eta^{-m},$$

where *β* is a constant, and *b* and *m* are scaling coefficients. Estimates of *b* range from 0.5 to 1 [25,38,42], and estimates of *m* range from 0.4 to 4 depending on the species [25], with a value of 1 found for *Chlamydomonas* [43]. Using the VFT equation to define viscosity and equation (2.10) to define velocity in equation (2.9) results in a model for ballistically motile resource uptake as a function of temperature and organism radius.

Organismal metabolism was modelled by employing the MTE (equation (2.2)) to model basal metabolism with a motility cost. The basal metabolism scales with organismal mass, which is proportional to the number of cells in the organism. Owing to its hollow-sphere morphology, the basal metabolic rate is proportional to organismal surface area:

2.11 $$B=B_{0}\;e^{-E_{a}/kT}4\pi Ra^{2}.$$

Assuming the organism exists at a Reynolds number less than 1 (i.e. where viscous forces of the fluid are dominant over inertial forces), the power it takes to maintain a velocity *v* through the fluid is given by Stokes’ Law [44], which, along with a coefficient of efficiency (ϵ), acts as the motility cost:

2.12 $$W=6\pi \frac{a\eta \rho }{\epsilon }v^{2}.$$

Incorporating each component of the model, the full energy balance becomes:

2.13 $$C_{\infty }\pi a^{2+b}\beta {\left(\eta_{0}\;e^{A/(T-C)}\right)}^{-m}\geq 4B_{0}\;e^{-E_{a}/kT}a^{2}R+W,$$

where *W*, the metabolic cost of motility, can be expanded using equations (2.10), (2.12) and the VFT equation to be a function of temperature and organism radius (see electronic supplementary material for complete expression).

## 3. Results

### (a) Global productivity model

**Figure 2.**
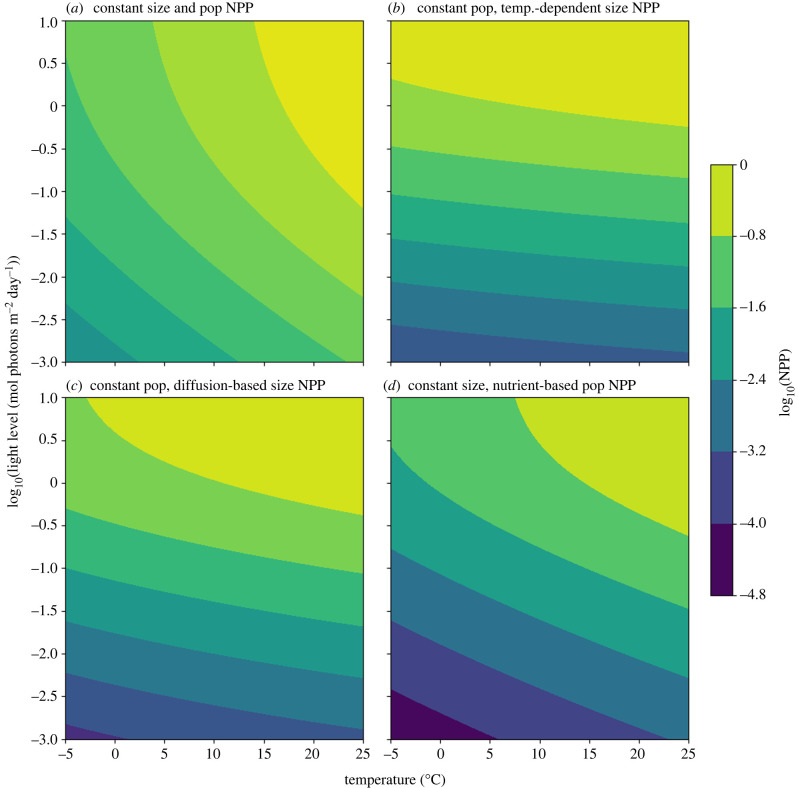
Contour-plots showing the log_10_ of net primary productivity (NPP) as a function of temperature (°C) (*x*-axis) and the relative log_10_ of photosynthetically active light availability (*y*-axis). (*a*) Global NPP given a constant number of primary producers with constant mass. (*b*) Global NPP given a constant number of primary producers, but their mass changes as a function of temperature based on the diffusion model (equation (2.8)). (*c*) Global NPP given constant population size, where the size of primary producers scales with the diffusion model (nutrient concentration is assumed to decrease with temperature, and is used to calculate producer size). (*d*) Global NPP where size is held constant, but population changes with temperature and limiting-nutrient concentration based on the steady-state biomass model in [33].

Four models of NPP were developed and analysed under varying ecological and physiological responses to environmental changes (figure 2; see electronic supplementary material for parameter table). Models were evaluated over the same range of temperature and PAR availability, but population size and producer size were either held constant or allowed to vary according to models.

Under the best case, where primary producer mass and population size each remain constant with decreasing temperature and light, reduced metabolic rates lead to a two order-of-magnitude decrease in NPP (figure 2*a*). In reality, most primary producers rely on diffusion to obtain the inorganic nutrients needed for growth. The diffusion model (equation (2.8)) can be employed to consider how the primary producer’s size would have changed as temperature decreased. Assuming that both the concentration of inorganic nutrients and the number of primary producers are constant, introducing the temperature–size dependence of the primary producers indicates that NPP would decrease by 2.5–3 orders of magnitude (figure 2*b*).

During the Cryogenian, environments capable of supporting life became more oligotrophic, reducing resource availability, and became eutrophic after melting [17,45]. The impact of nutrient availability was incorporated into the NPP model by assuming that nutrient availability linearly decreases by half over the temperature interval. Nutrient availability could impact the size of primary producers (figure 2*c*) or the number of primary producers (figure 2*d*). Both cases lead to significant decreases in NPP, with an approximately 3.5 order-of-magnitude decrease for nutrient-limited cell size, and a 4.5 order-of-magnitude decrease for nutrient-limited population size. Even when assuming resilient physiologies and ecosystems, decreased organic resource availability would have been a major environmental change for existing heterotrophic organisms.

### (b) The diffusive cell

**Figure 3.**
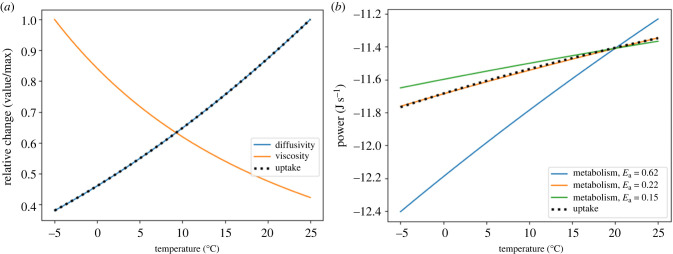
(*a*) Relative changes (value/max) in viscosity, diffusivity and uptake rate for the diffusive cell as functions of temperature (°C). (*b*) Value of uptake rate and metabolic rate (log_10_*W*) of the diffusive cell as functions of temperature (°C). (*b*). Metabolic rate is plotted for three different *E*_a_ values.

The non-motile diffusive cell’s dependence on temperature (equation (2.8)) is twofold: (i) the metabolic rate’s dependence on temperature and (ii) the uptake rate’s dependence on diffusivity and viscosity. The decrease in temperature that accompanied Snowball Earth caused an increase in viscosity accompanied by a decrease in diffusivity and nutrient uptake, but also led to a slower metabolic rate. Although uptake drops to less than half of its pre-Snowball Earth value, under an activation energy of 0.62 eV, metabolic rate drops by nearly a factor of 10 (figure 3). The slow-down in metabolic rate means that, although the cell’s uptake slows, it is able to grow in size as temperature decreases.

**Figure 4.**
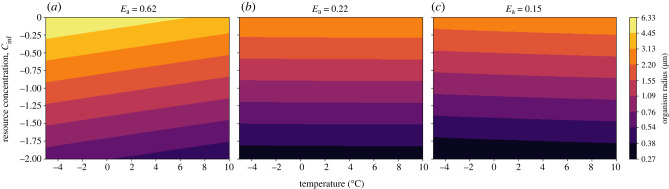
Contour-plot of the log of radius ($\log_{10}a$) of the diffusive cell as a function of temperature (°C) (*x*-axis) and relative resource concentration (*y*-axis). Each subplot shows the results under a different activation energy (*E*_a_).

Based on the results from the NPP calculation, it is important to consider a decrease in organic resource concentration in addition to temperature decrease during Snowball Earth. The non-motile organism relying on diffusion must shrink in size, reducing its radius *a*, to adapt to lower resource availability (figure 4). Under the best supported parameter values (see electronic supplementary material), the model predicts a cell radius of approximately 6 μm prior to Snowball Earth and a radius of approximately 0.25 μm during Snowball Earth. Importantly, we show that cell size changes are greatly impacted by the assumed value of average metabolic activation energy *E*_a_. This value influences how metabolism scales with temperature, impacting the relative change between uptake and metabolic rate (figure 3*b*). For all values of *E*_a_, there is a decrease in cell size as resource availability drops, but varying values of *E*_a_ can change the temperature dependence of diffusive cell size (figure 6). While the average metabolic activation energy determines the response to temperature, all diffusive organisms, regardless of *E*_a_, must have decreased in size to survive the Cryogenian period owing to the decrease in resource availability.

### (c) The motile choanoblastula

The choanoblastula’s motility introduces an additional temperature dependence to the energy balance owing to the cost of motility’s dependence on viscosity (*η*) of water. However, the motility cost is relatively small compared with the basal metabolic cost and uptake rate, and therefore has a negligible effect (figure 7). Resource uptake scales with organism radius (*a*^1+2*b*^, where 0.5 ≤ *b* ≤ 1) more quickly than the metabolic rate, which scales with *a*^2^ owing to cells only existing on the sphere’s surface. Because resource uptake scales at a higher rate, there exists a critical size where for smaller radii the metabolic rate is greater than the uptake rate, and for larger radii the uptake rate is greater than the metabolic rate (figure 7). This critical radius defines the minimum size of the organism for the given temperature and resource concentration, and is the solution to the energy balance in equation (2.13).

**Figure 5.**
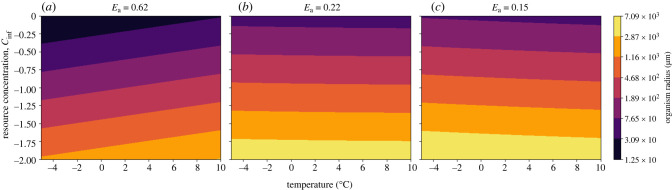
Contour-plot of the log of radius $\log_{10}a$ of the choanoblastula as a function of temperature (°C) (*x*-axis) and relative resource concentration (*y*-axis). Each subplot shows the results under a different activation energy (*E*_*a*_). Plots are for *b* = 1.

**Figure 6.**
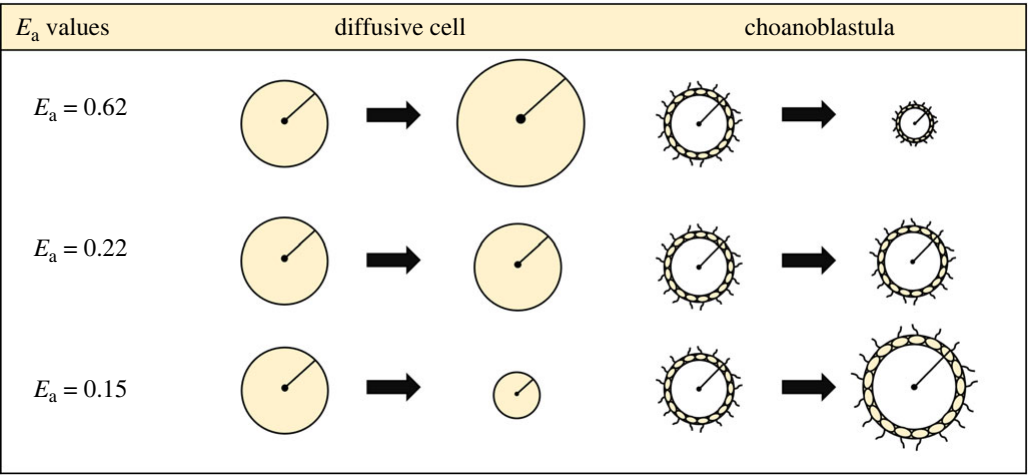
Summary of organism size dependence on temperature for the diffusive cell and the choanoblastula with differing *E*_a_ values under constant resource concentration.

The critical radius increases with decreasing nutrient concentration, suggesting organisms using this strategy would have increased in size in response to the environmental changes during Snowball Earth (figure 5). Under the best estimates for parameter values, the choanoblastula goes from a minimum radius of approximately 12.5 μm prior to Snowball Earth to a minimum radius of approximately 7 mm during Snowball Earth. Like the diffusive model, the activation energy *E*_a_ impacts the relationship between temperature and organism size. While an activation energy of 0.62 eV results in size decreasing with decreasing temperature, an activation energy below 0.22 eV inverts the relationship (figure 6). Regardless of average activation energy, the choanoblastula would have increased in size during Snowball Earth owing to the drop in resource availability.

## 4. Discussion

### (a) Ecological changes during Snowball Earth

Changes in temperature, inorganic nutrient concentrations and light availability had major impacts on the existing organisms and broader ecosystem. The exponential dependence of metabolic rate on temperature caused the primary producer metabolic rates to decrease with temperature, slowing productivity. This decrease was further exacerbated by the physiological and ecological impacts caused by the physical changes accompanying the onset of Snowball Earth glaciations, including reduced light under sea ice, higher viscosity and lower diffusivity. Under the most conservative assumption that primary producer size and population did not change, NPP would still decrease by two orders of magnitude (figure 2*a*). When the impacts of both nutrient concentration and temperature are considered, that decrease varies between 2.5 and 4.5 orders of magnitude (figure 2*b*–*d*).

A reduction in NPP of this magnitude would pose a significant hurdle for heterotrophs, leading to an increase in competition for the remaining resources. This increase in competition was a significant evolutionary driver, which may help to explain why multiple multicellular lineages appeared in this time frame. The diverging response of the two modelled organisms shows two possible evolutionary paths. Heterotrophic eukaryotes in the Cryogenian were forced to either get smaller and compete with prokaryotes better suited to the diffusive regime, or become larger, more complex and multicellular. These observed alternative strategies help explain why some, but not all, eukaryotes evolved multicellularity during this time.

### (b) Morphological differences lead to different adaptive strategies

A key difference between the two presented morphological models is the scaling between organism size and uptake that originates from two mechanistically different uptake strategies. In the diffusive model, uptake scales with organismal radius owing to the physics of diffusion constraining its rate (equation (2.6)). By becoming motile and entering the ballistic regime, the choanoblastula uptake rate scales with its cross-sectional area (equation (2.9)) and its velocity (equation (2.10)), which in turn scales with organism size. This difference means that an increase in size leads to a large increase in uptake for the choanoblastula compared with the diffusive cell.

Bacterial multicellularity is common and diverse, with quorum sensing, metabolic division of labour, large size and spatial structure [46–50]. In particular, stromatolites have a deep geological history, potentially extending back to the first fossil evidence of life [51,52]. As all bacteria are obligatory diffusion specialists, life within a stromatolite is subject to the same physical processes we model for a solitary diffusive cell [53,54]. Therefore, we can make a first-order prediction that the effects of Snowball Earth conditions on stromatolites should match the predictions for solitary diffusive cells. This may provide an additional prediction for the decline in stromatolite abundance and size in the late Neoproterozoic prior to the origin and diversification of grazing and bioturbating bilaterian animals [55,56].

**Figure 7.**
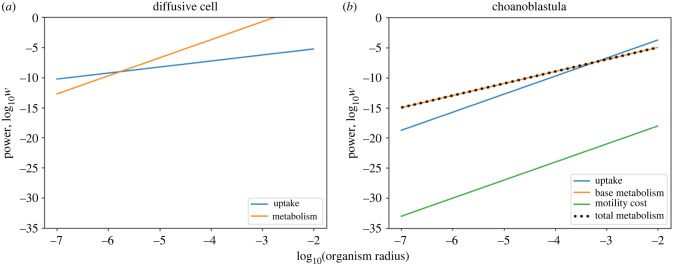
Energetic costs and nutrient uptake as a function of organism radius for (*a*) the diffusive cell and (*b*) the choanoblastula models (based on a temperature of 0°C and nutrient concentration *C*_∞_ = 0.1).

At the size of eukaryotic cells and simple metazoa, the cost of motility becomes vanishingly small, and provides an enormous benefit for maintaining a larger size by increasing resource uptake (figure 7*b*). However, becoming motile is not enough to offset lower resource availability. The hollow morphology is essential, as it reduces the mass-scaling of metabolic cost of the organism by reducing metabolically active volume while maintaining effective surface area for nutrient uptake. This change in scaling is ubiquitous among complex multicellular organisms, as seen in the infamous two-thirds and three-quarter power laws [29].

Together, these adaptations invert the relationship between nutrient uptake and metabolic rate as a function of organism size. For the diffusive cell, metabolic rate increases faster than uptake, constraining the maximum cell size (figure 7*a*). The opposite is true for the choanoblastula, in which faster uptake means that the energy balance defines a minimum size, allowing it to grow larger until other constraints are reached (figure 7*b*) [41].

### (c) Adaptation of activation energy

Activation energy (*E*_a_) is the amount of energy required to reach a transition state, and the source of this energy required to drive reactions is typically heat energy from the surroundings. These results show that organismal size responses to changes in temperature are highly sensitive to activation energy (figures 4 and 5). Activation energies vary significantly across life on Earth [57], although much research assumes an average value (0.62 eV [58]); assuming this value in our models (and thus constraining the relationship between metabolic rate and resource uptake to a specific regime) suggests that diffusive cells must get larger at lower temperatures and the choanoblastula organisms must get smaller (figure 6).

However, given the range of measured activation energies, and the fact that unicellular organisms commonly display lower average energies [57], it is necessary to consider differential relationships between metabolic rate and nutrient uptake. The metabolic activation energy emerges from the average activation energies of the underlying enzyme-catalysed reactions that fuel the organism’s metabolism. Over the 50 Myr glacial period, it is possible that organisms were selected to have lower activation energies in order to maintain their metabolisms at lower temperatures. At an activation energy of 0.22 eV, the body size for both morphological models no longer varies with temperature, and the body size–temperature relationship becomes inverted for both models when the activation energy is less than 0.22 eV. These inversions coincide with the difference in slopes of metabolism under each activation energy relative to the nutrient uptake rate (figure 3). Determining the adaptability of metabolic activation energy would be an important step to understanding the range of possible evolutionary trajectories in changing climates.

### (d) Pre- and post-Snowball dynamics

**Figure 8.**
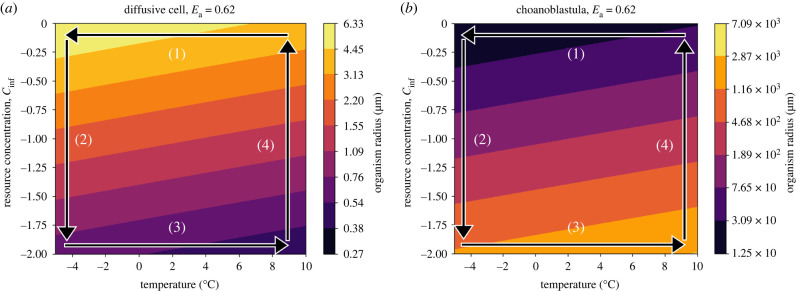
log_10_(radius) of (*a*) the diffusive cell and (*b*) the choanoblastula, shown as contour plots as functions of temperature and resource concentration. Labelled arrows represent possible trajectories in temperature–resource concentration space for the onset (arrows 1 and 2) and termination (3 and 4) of Snowball Earth.

The paths taken through temperature–resource concentration space during the onset and termination of the Cryogenian glacial periods are important to consider in order to understand the evolutionary trajectories of the existing organisms. Given that primary production decreases owing to decreasing temperature and PAR availability, it is likely that temperature decreased faster than resource availability during glacial onset. This trajectory causes diffusive cells to initially grow, reaching their maximum predicted size (approx. 6 μm) while the choanoblastula reach their minimum (approx. 12 μm) (figure 8, arrow 1). This places the two modelled organisms in a remarkably similar size range, with radii less than an order of magnitude apart, and at around 10 μm, approximately the size of a modern *Chlamydomonas* [59] or *Salpingoeca* cell [60]. Then, as resource concentrations begin to drop, the organisms’ evolutionary pathways diverge as the diffusive cell is forced to shrink and the choanoblastula grows (figure 8, arrow 2).

Following Snowball Earth glaciations, temperature and resource availability increased. Like the onset, it is likely that temperature rebounded before resource concentrations rose. As temperature increased and NPP rates had not yet recovered, choanoblastula would continue to get larger, reaching the maximum predicted size, as the diffusive cell reaches its minimum (figure 8, arrow 3). As resource concentrations rise, the model predicts that the choanoblastula would shrink and the diffusive cell would grow (figure 8, arrow 4). While it is likely that some organisms reduced their size in response, the larger size, accompanied by a now increasing amount of resources and faster metabolic rates, could allow new ecological strategies such as predation to develop, permitting the organism to maintain its size as resource availability continues to increase. The hysteretic difference in trajectories through nutrient–temperature space, as well as the new ecological selective pressures arising at the end of the glaciation periods, may help to explain the rapid proliferation of macroscopic fossils and early metazoan lineages that appear in the Ediacaran.

## 5. Conclusion

A variety of hypotheses have been proposed to explain the propensity of eukaryotic lineages to evolve complex multicellularity more readily: the enhanced energy capacity provided to eukaryotes by mitochondria [61], the eukaryotic-style ‘default off’ mode of gene regulation [62], the differential effects of genetic drift on prokaryotic and eukaryotic genomes [63], and the significance of the nucleus in separating transcription and translation [64]. While each of these features plays an important role in differentiating eukaryotic and prokaryotic multicellularity, none provides a definitive answer for the 1.5 billion year gap between eukaryogenesis and the emergence of complex multicellular lineages, or for why multiple eukaryotic clades evolved multicellularity during the Neoproterozoic [65]. The Neoproterozoic Snowball Earth glacial events provide an environmental driver that our models show would have selected for multicellular morphologies during this time period, helping explain the lag between eukaryogenesis and the proliferation of complex multicellularity.

The mechanistic models presented were chosen for their resemblance to what we consider to be likely morphologies during this period, namely diffusive prokaryotic cells and simple, motile multicellular organisms similar to a hypothetical early metazoan lineage. While the presented models do not consider the increase in regulatory and signalling machinery necessary to maintain a complex multicellular organism, they show that a multicellular morphology is adaptable from an energetic perspective [66,67]. More generally, the argument presented comes down to differences in scaling between nutrient uptake and metabolic rate under changes in an organism’s environment. The hollow sphere represents one possible early morphology, and is not likely to be a realistic morphology for all the ancestors of complex multicellular lineages. While it may represent only one of many different possible optima, the general scaling patterns may be more universal. The transition to multicellularity is accompanied by a transition to sublinear scaling of metabolic rates, where metabolism was likely proportional to surface area prior to the evolution of circulatory systems [30,31]. However, additional work to quantify how alternative morphologies of early multicellular and colonial organisms may have responded under these conditions will be an important next step to understand how generalizable these scaling features are [68]. Do the stationary branching body-plans observed in Ediacaran rangeomorphs or the branching canals of demosponges offer alternative adaptive paths to Snowball Earth conditions [69,70]? Additional analysis of the biophysical constraints of a variety of morphologies could further support Snowball Earth as an environmental trigger for the proliferation of complex multicellularity across several eukaryotic clades.

Our finding that the adaptive strategies arising during the cold, highly viscous and nutrient-poor conditions of global glaciations are shaped by metabolic scaling and the method of resource acquisition offers a potential explanation for the differences in the types of multicellularity between bacteria and eukaryotes. Bacteria are obligate osmotrophs owing to the constraints on their cell size arising from their scaling features, including the superlinear scaling of their metabolism to mass and ribosomes to volume [31,71]. With the inability to escape the diffusive regime, they were constrained to decrease in size. Instead, eukaryotic cells, which were already able to reach greater size owing to their linear scaling (likely owing to mitochondria) would have been able to transition into the ballistic uptake regime. While the temperature model suggests that diffusive cells may initially get larger as temperature decreases, prokaryotes face fundamental barriers in their size owing to scaling that eukaryotes do not. The Cryogenian glaciations therefore provided an opportunity for multicellular eukaryotes to have a selective advantage that bacteria do not share.

Studies of choanoflagellates suggest that they live individually in low-resource regions but form colonies at high nutrient concentrations, while studies of *Trichodesmium* suggest that colony formation allows them to sink faster to reach nutriclines [72,73]. These observations also call for an analysis of the selective effect of resource patchiness as an additional environmental feature in the evolution of multicellularity, and the potential differences in the function of multicellular motility between prokaryotes and eukaryotes.

## Data Availability

The data are provided in the electronic supplementary material [74].
